# CpG oligodeoxyribonucleotides protect mice from *Burkholderia pseudomallei *but not *Francisella tularensis *Schu S4 aerosols

**DOI:** 10.1186/1476-8518-8-2

**Published:** 2010-02-05

**Authors:** David A Rozak, Herbert C Gelhaus, Mark Smith, Mojgan Zadeh, Louis Huzella, David Waag, Jeffrey J Adamovicz

**Affiliations:** 1 Bacteriology Division, United States Army Medical Research Institute of Infectious Diseases, Fort Detrick, Maryland, USA; 2 Pathology Division, United States Army Medical Research Institute of Infectious Diseases, Fort Detrick, Maryland, USA; 3 Battelle Biomedical Research Center, Battelle Memorial Institute, West Jefferson, Ohio, USA; 4 Department of Medicine, Feinberg School of Medicine, Northwestern University, Chicago, Illinois, USA; 5 Center for Biological Safety and Security, Midwest Research Institute, Frederick, Maryland, USA

## Abstract

Studies have shown that CpG oligodeoxyribonucleotides (ODN) protect mice from various bacterial pathogens, including *Burkholderia pseudomallei *and *Francisella tularensis *live vaccine strain (LVS), when administered before parenteral challenge. Given the potential to develop CpG ODN as a pre-treatment for multiple bacterial biological warfare agents, we examined survival, histopathology, and cytokine data from CpG ODN-treated C57BL/6 mice to determine whether previously-reported protection extended to aerosolized *B. pseudomallei *1026b and highly virulent *F. tularensis *Schu S4 infections. We found that, although CpG ODN protected mice from aerosolized *B. pseudomallei *challenges, the immunostimulant failed to benefit the animals exposed to *F. tularensis Schu S4 *aerosols. Our results, which contrast with earlier *F. tularensis *LVS studies, highlight potential differences in *Francisella *species pathogenesis and underscore the need to evaluate immunotherapies against human pathogenic species.

## Findings

Bacteria-derived CpG oligodeoxyribonucleotides (ODN) are potent stimulators of the innate immune system, acting via the toll-like receptor (TLR) 9 signaling pathway. Consequently, the small CpG-rich motifs [1] have been successfully developed as adjuvants for a broad array of bacterial subunit vaccines and are currently undergoing multiple clinical trials [2]. CpG ODNs have also been administered alone as pretreatments to effectively protect mice from infection by different bacterial pathogens [3], including the biological warfare threat agents *Burkholderia mallei *[4] and *Burkholderia pseudomallei *[5]. Murine studies of the *Francisella tularensis *live vaccine strain (LVS) [3] suggest that CpG ODN may also protect against human-virulent *F. tularensis *Schu S4 infection. Significantly, researchers have shown that CpG ODNs protect mice from *F. tularensis *LVS when the therapy is administered anywhere from two days to two weeks before parenteral challenge [3] and can maintain this protection indefinitely when CpG ODN is given two to three times a month [6]. Provided CpG ODNs can broadly protect against different aerosolized biological warfare agents days or weeks after treatment, the molecules may represent potential pretreatments for military or civilian personnel at risk of exposure to the weaponized bacteria.

In order to determine whether CpG ODN protects mice from aerosolized *B. pseudomallei *1026b or *F. tularensis *Schu S4 challenges (all previous reports deal with parenteral challenges), we gave groups of 8-10 anaesthetized 8-week-old female C57BL6/J mice 20 μl i.n. or 200 μl i.p. injections of Hank's basal salt solution (HBSS) 48 h before or 1 h after receiving aerosolized doses of *B. pseudomallei *1026b or *F. tularensis *Schu S4.

Depending on the group, 150 μg CpG ODN 10103 (Coley Pharmaceutical Group: 5'-TCG TCG TTT CGT CGT TTT GTC GTT-3'), which has been developed as a potent stimulant for the human immune system, was added to the saline treatment. CpG ODN variants are known to produce different immunostimulatory responses in different mammalian species [7]. However, our own unpublished studies suggest that CpG ODN 10103 performs comparably in mice to CpG ODN 7909 (5'-TCGTCGTTTTGTCGTTTTGTCGTT-3'), which was previously reported to protect the BALB/c mice from *Burkholderia mallei *challenges [4]. Despite CpG ODN 10103's traditional use as a human adjuvant, we have found that the immunostimulant's ability to protect mice from aerosolized *B. pseudomallei *(reported here) and *B. mallei *(unpublished data) suggest that, as with the CpG motifs used in other *Burkholderia *and *F. tularensis *murine studies [3-6], the oligodeoxyribonucleotide is an effective immunostimulant in both C57BL6/J and BALB/c mice.

**Figure 1:**
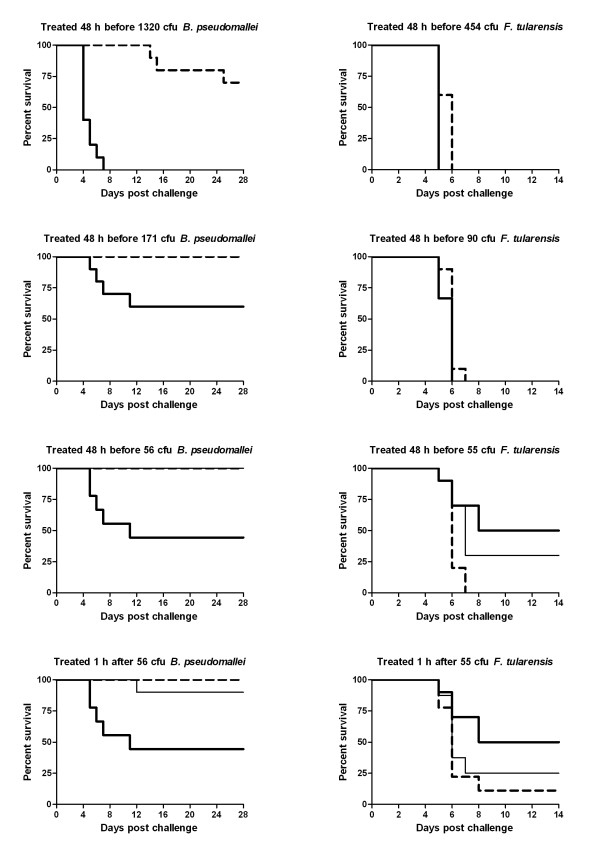
**CpG ODN 10103 differently protects mice from aerosolized *B. pseudomallei *1026b and *F. tularensis *Schu S4 challenges**. Groups of 8-10 C57BL6/J mice were given saline (bold solid lines) or 150 μg CpG ODN 10103 via intranasal (bold dashed lines) or intraperitoneal (thin solid lines) injection 48 h before or 1 h after being challenged with aerosolize *B. pseudomallei *1026b or *F. tularensis *Schu S4 and observed for 14-28 days. The calculated inhaled bacterial doses are given above each graph. Intraperitoneal and 1 h post-challenge therapies were only administered during the low-dose challenges. Survival curves were compared using log-rank (Mantel-Cox) tests.

We gave saline control and CpG ODN 10103-treated mice different aerosolized doses of *B. pseudomallei *1026b or *F. tularensis *Schu S4 prepared from 37°C overnight glycerol tryptone broth or IsoVitaleX (BD Diagnostic Systems, Sparks, MD)-supplemented Mueller Hinton broth cultures, respectively. Serial dilutions of nebulizer and impinger medium were prepared in triplicate and spread on solid media to calculate actual inhaled doses. When necessary, mice from each treatment group were split evenly between consecutive aerosol runs to accommodate potential variations in aerosol dose from one run to the next. As indicated in Figure 1, CpG ODN-treated mice were significantly protected from high and low doses of aerosolized *B. pseudomallei *1026b challenges (p < 0.05 when compared to saline controls). However this was not the case for *F. tularensis *Schu S4-infected animals, which were unprotected regardless of challenge dose or treatment route. In fact, our data suggests that CpG ODN 10103 therapies may have adversely affected the survival of *F. tularensis*-infected mice. Specifically, mice given 150 μg CpG ODN i.n. 48 h prior to challenge actually exhibited a statistically significant (p < 0.025) decrease in survival when compared to controls. Furthermore, when all mice receiving 55 cfu aerosolized *F. tularensis *Schu S4 are grouped according to whether or not they received CpG ODN, regardless of treatment route and time, the untreated controls exhibited a statistically elevated survival curve (p = 0.05). *B. pseudomallei *and *F. tularensis*-infected mice were statistically unaffected by giving the CpG ODN treatments via different routes (i.n. or i.p.) and times (48 h before or 1 h after infection).

**Figure 2:**
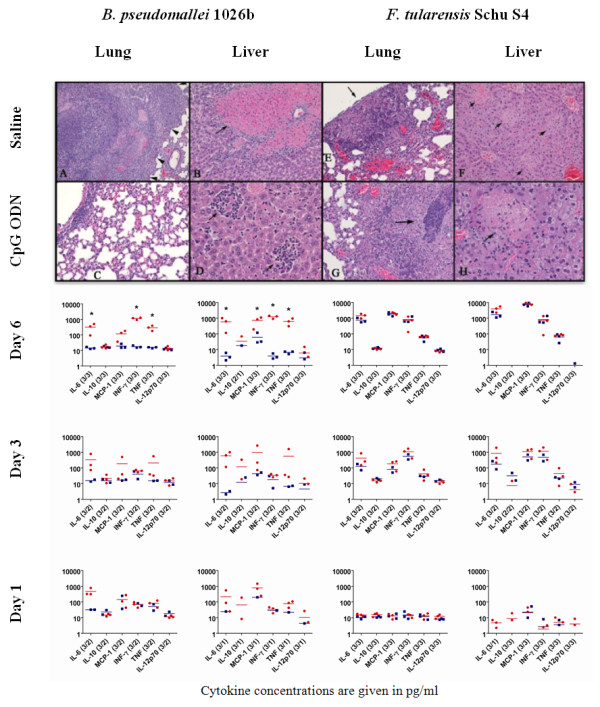
**CpG ODN 10103 administered intranasally 48 h prior to infection differently effects *B. pseudomallei *1026b and *F. tularensis *Schu S4 pathogenesis in aerosol-challenged mice**. At 6 days post infection, mice challenged with *B. pseudomallei *developed bronchopneumonia and pulmonary abscesses (A: arrowheads) and areas of liver necrosis (B: arrows). By contrast, *B. pseudomallei*-infected mice pretreated with CpG ODN developed only mild interstitial pneumonia (C) and lymphoplasmacytic hepatitis (D: arrows). *F. tularensis*-challenged mice exhibited bronchopneumonia and pulmonary abscesses (E, G: arrows) and areas of liver necrosis (F, H: arrows) 6 days after infection regardless of whether or not they received CpG ODN. Cytokine levels in the livers and lungs of saline (red circles)- and CpG ODN (blue squares)-treated mice differed significantly for mice infected with *B. pseudomallei *but not *F. tularensis*. Mean cytokine levels for tissues obtained from mice 1, 3, and 6 days after infection are rendered as colored bars. Asterisks indicate statistically significant differences (p < 0.05) between saline- and CpG ODN-treated groups on day 6. Parenthetical values accompanying cytokine labels indicate numbers of independent data points obtained for saline- and CpG ODN-treated mice. Cytokine concentrations below 1 pg/ml fall below the x-axis and are not shown on the graphs. T-tests were used to support qualitative analysis of CBA-generated cytokine data.

To further explore the bacterial response to CpG ODN treatments, we repeated aerosol challenges for groups of nine mice that had received CpG ODN i.n. treatments 48 h before infection. Each of the mice received average inhaled doses of 236 cfu of *B. pseudomallei *1026b or 3,100 cfu of *Francisella *Schu S4. Three mice from each experimental group were euthanized 1, 3, and 6 days post infection to observe disease progression in lungs, livers, and spleens. In a qualitative review of hematoxylin- and eosin-stained, paraffin-embedded tissues, CpG ODN-treated mice exhibited elevated levels of hepatitis and interstitial pneumonia at all time points compared to untreated mice, regardless of whether or not they were challenged with *B. pseudomallei*1026b or *F. tularensis *Schu S4. The latter observation is consistent with CpG ODN-induced lung inflammation previously reported for uninfected mice [8]. Whereas CpG ODN treatment prevented bronchopneumonia, pulmonary abscesses, liver necrosis, and splenic thrombi in *B. pseudomallei *1026b-infected mice 3 and 6 days postinfection, the development of pulmonary abscesses and hepatic and splenic necrosis in *F. tularensis *Schu S4-challenged mice remained unaffected by CpG ODN treatment. Six days postinfection, *B. pseudomallei *and *F. tularensis *infections of untreated mice were typified by bronchopneumonia and thrombosis in the former and interstitial pneumonia, hepatitis, and splenic necrosis in the latter (Figure 2).

Tissue samples from the same mice were also ground and diluted 10-fold by mass into 25 mM pH 7.0 potassium phosphate buffer before aliquots were sampled using the BD Cytokine Bead Array (CBA) Mouse Inflammation kit (BD Biosciences, San Jose, CA) according to the manufacturer's protocol. Cytokine levels in the livers, lungs, and spleens of mice euthanized on days 1, 3, and 6 postinfection correlated well with the *B. pseudomallei*- and *F. tularensis*-induced pathogenesis observed in the same tissue samples (Figure 2). Whereas interleukin (IL)-6, monocyte chemotactic protein (MCP)-1, interferon (IFN)-γ, and tumor necrosis factor (TNF) were statistically elevated in the hepatic tissue of untreated *B. pseudomallei*-infected mice, the cytokines dropped to basal levels in the lungs and livers of similarly infected CpG ODN-treated mice, which generally lacked culturable bacteria at 6 days postinfection. Conversely, 6 days after infection, cytokine levels were dramatically elevated in the lungs, livers, and spleens of *F. tularensis*-challenged mice, regardless of whether or not they received CpG ODN treatments. Elevated cytokine levels in the lungs and spleens of *F. tularensis*-infected animals 6 days postinfection correlated well with the recruitment of immune cells to the interstitial lung tissue and acute necrosis of the spleen observed by histopathology.

The novel aerosol data presented here corroborate previous reports of CpG ODN-mediated protection for *B. pseudomallei*-infected BALB/c mice with novel data for low- and high-dose aerosolized challenges of C57BL6/J mice. However, the results of our *F. tularensis *Schu S4 experiments contrast with earlier accounts of the protection offered by CpG ODN treatments for C57BL6/J mice challenged with the less virulent *F. tularensis *LVS. The independent survival and histopathology experiments presented in Figures 1 and 2 reveal that CpG ODN 10103 failed to positively affect the course of *F. tularensis *Schu S4 pneumonic infection and statistically reduced the survival rates for CpG ODN-treated mice exposed to low-dose *F. tularensis *Schu S4 aerosols.

Differing responses to immunostimulatory treatments have already been reported among *Francisella *species and appear to be consistent with the potentially adverse effects observed here for *F. tularensis *Schu S4, which is known to actively impair host immune response [9,10]. Specifically, Kieffer et al reported that mice treated with either *F. tularensis *LVS- or *F. novicida *U112-derived lipopolysaccaride (LPS) 3-5 days before bacterial challenge were protected from *F. tularensis *LVS, but not *F. novicida *U112, infections [11]. In fact, the group observed that their LPS therapies may have actually enhanced the virulence *F. novicida*, which is more closely related by genome analysis [12,13] and murine virulence models [11] to *F. tularensis *type A strains than type B. Therefore, while our findings regarding *F. tularensis *Schu S4 infection in CpG ODN-treated mice are novel, they are not entirely unexpected in the context of *Francisella *research and further underscore the need for immunotherapeutic studies in both A and B subtypes.

Finally, we would like to assert that all research was conducted in compliance with the Animal Welfare Act and other federal statutes and regulations relating to animals and experiments involving animals and adheres to principles stated in the Guide for the Care and Use of Laboratory Animals, National Research Council, 1996. The facility where this research was conducted is fully accredited by the Association for Assessment and Accreditation of Laboratory Animal Care International.

## Abbreviations

CBA: cytokine bead array; CFU: colony forming unit; HBSS: Hank's basal saline solution; IFN: interferon; IL: interleukin; LPS: lipopolysaccaride; LVS: live vaccine strain; MCP: monocyte chemotactic protein; MTM: modified Thayer-Martin agar; ODN: oligodeoxyribonucleotide; TLR: Toll-like receptor; TNF: tumor necrosis factor.

## Competing interests

The authors declare that they have no competing interests.

## Authors' contributions

DAR and HCG oversaw the general design, implementation, and analysis of the experiments described in this manuscript. MS and LH provided expert analysis of paraffin-embedded tissue samples. MZ contributed the CBA analysis of murine cytokine levels. DW and JJA offered critical guidance and oversight for the project. All authors have read and approve of this manuscript.
